# Antimicrobial Resistance of *Listeria monocytogenes* from Animal Foods to First- and Second-Line Drugs in the Treatment of Listeriosis from 2008 to 2021: A Systematic Review and Meta-Analysis

**DOI:** 10.1155/2022/1351983

**Published:** 2022-10-07

**Authors:** Jaqueline Oliveira Dos Reis, Bruno Serpa Vieira, Adelino Cunha Neto, Vinicius Silva Castro, Eduardo Eustáquio de Souza Figueiredo

**Affiliations:** ^1^Faculty of Agronomy and Animal Science, Federal University of Mato Grosso, Cuiabá 78060-900, Brazil; ^2^Faculty of Animal Science, Federal Institute of Mato Grosso, Alta Floresta 78580-000, Brazil; ^3^Faculty of Nutrition, Federal University of Mato Grosso, Cuiabá 78060-900, Brazil; ^4^Department of Biological Science, University of Lethbridge, ULETH, Lethbridge, Canada

## Abstract

First-line drugs for the treatment of listeriosis are the same around the world, but particular conditions might reduce their efficacy, including antimicrobial resistance. Therefore, this study aimed to verify, based on a systematic review and meta-analysis, whether the prevalence of antimicrobial resistance in *Listeria monocytogenes* from animal foods is higher for first- or second-line antimicrobials. From the total of 302 identified studies, 16 met all the eligibility criteria from 2008 to 2021 and were included in this meta-analysis. They comprised a dataset of 1152 *L. monocytogenes* isolates, obtained from different animal food products, food processing environment, and live animals. The included studies were developed in South America (*n* = 5), Europe (*n* = 4), Asia (*n* = 3), Africa (*n* = 2), and North America (*n* = 2), testing a total of 35 different antimicrobials, 11 of them classified as first-line drugs. Complete lack of antimicrobial resistance across the studies (all *L. monocytogenes* isolates tested as susceptible) was only observed for linezolid, while widespread antimicrobial resistance (all *L. monocytogenes* isolates tested resistant) was described for amoxicillin, benzylpenicillin, cefoxitin, fusidic acid, imipenem, sulfamethoxazole, and vancomycin. Overall, the meta-analysis results indicated no evidence that antimicrobial resistance in *L. monocytogenes* isolated from animal-based food is higher for first-line antimicrobials compared to second-line compounds (*p*=0.37). A greater volume of publication, together with better characterization of the isolates, is still needed for a more precise estimate of the real prevalence of antimicrobial resistance in *L. monocytogenes*.

## 1. Introduction

*Listeria* genus comprises of twenty-one species, such as *L. grayi*, *L. innocua*, *L. ivanovii*, *L. monocytogenes*, *L. seeligeri,* and *L. welshimeri*, among others [1]. Among them, only *L. monocytogenes* (*Listeria monocytogenes*) is considered pathogenic to humans and other animals [2] being implicated in severe cases of sepsis, encephalitis, meningitis, and abortions, with high rates of hospitalization and death [3]. That is why the ability of *Listeria* to persist in food processing environments and multiply at low temperatures represents a public health concern. Not surprisingly, the number of recalls related to *Listeria* in the food industry is significant, especially in animal-based products such as beef, chicken, seafood, and dairy products [4].

The use of antimicrobials is the main strategy to treat infections and control food-borne illnesses caused by *L. monocytogenes*. However, the indiscriminate or inappropriate use of these molecules can lead to serious consequences that include the emergence of resistant strains [5].

The first-line drugs to treat listeriosis are ampicillin and penicillin G (beta lactams), frequently associated with gentamicin or another aminoglycoside. The combination of trimethoprim and sulfamethoxazole (folate pathway inhibitors) is also recommended, especially to patients with allergy to beta lactams [6, 7]. In addition to those first-line drugs, other molecules such as erythromycin (macrolides) and tetracyclines may be used [8]. Despite their importance in situations where first-line drugs are prohibitive, those molecules are frequently referred as second-line drugs.

Because antimicrobial resistance is a threat to the success of listeriosis treatment, it is believed that the choice of a therapeutic protocol must be supported by robust scientific evidence. In this sense, understanding the current phenotypic profile of antimicrobial resistance in *L. monocytogenes* isolated from different sources is of paramount importance. Therefore, this study was carried out to verify, based on a systematic review and meta-analysis, whether the prevalence of antimicrobial resistance in *L. monocytogenes* isolated from animal foods is higher for first- or second-line antimicrobials.

## 2. Materials and Methods

### 2.1. Search Strategy and Screening Process

Following the PICO strategy (participants, intervention, comparison, and outcomes) to organize search terms, a set of more than 50 Mesh descriptors and keywords were combined and tested in the Pubmed database. By excluding those that did not contribute in retrieving meaningful results, the following search algorithm was defined: (*Listeria monocytogenes*) AND (antimicrobial drug resistance) AND (beta lactams OR aminoglycosides OR trimethoprim OR sulfamethoxazole). This full algorithm was applied in the electronic databases such as Pubmed, Scielo, Science Direct, and Scopus, at the reference date of 15/11/2021. Electronic search was limited to titles, abstracts, and keywords. Only primary studies published in scientific journals were searched, once in literature reviews, meta-analysis, conference papers, books, and other bibliographic references were not desired.

**Figure 1:**
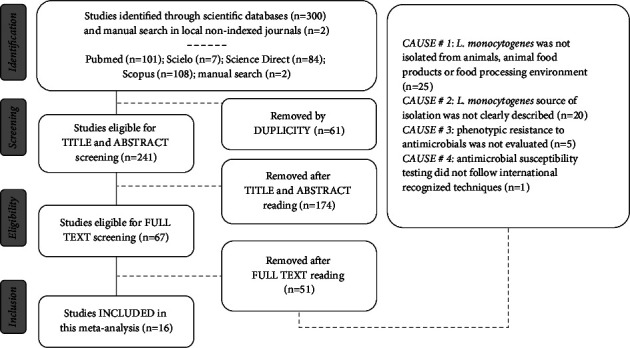
Flowchart of the systematic review procedure. Studies excluded by CAUSE # 1 [2–4, 6, 7, 51–55, 58–71]. Studies excluded by CAUSE # 2 [33, 34, 10–27]. Studies excluded by CAUSE # 3 [28–32]. Studies excluded by CAUSE # 4 [35].

All the search results were exported to a reference manager (EndNote, Clarivate™) where a screening process was conducted, excluding studies first by duplicity (automatically), and then after the sequential reading of titles, abstracts and full texts. In the end, only studies that met all the following eligibility criteria were included in this meta-analysis: (1) primary studies published in English; (2) compared the resistance of *L. monocytogenes* isolated from animals, animal food products or food processing environments to first- and second-line antimicrobials in the treatment of listeriosis; (3) adopted internationally recognized techniques (broth microdilution, disk diffusion, or Vitek-2) as antimicrobial susceptibility testing methodologies. A comprehensive illustration of the search and screening processes is presented in Figure1.

### 2.2. Data Extraction and Management

Data of phenotypic antimicrobial resistance (total number of analyzed and resistant *L. monocytogenes* isolates) were individually extracted from the included studies and organized in an electronic spreadsheet. The information was coded so that the study of origin, location (country), antimicrobials tested, animal chain (beef, chicken, dairy products, fish, or pork), and source of *L. monocytogenes* isolation (live animal, food processing environment, or food products) could be accessed later. As expected, several combinations of these classification items were obtained from each individual study.

For comparison purposes, antimicrobials were then grouped into two categories [8]: (1) first-line drugs—those classified as aminoglycosides, beta lactams, and folate pathway inhibitors; (2) second-line drugs—those in any other classification. A complete description of all the molecules assessed in this meta-analysis is presented in Table 1.

### 2.3. Meta-Analysis and Presentation of Results

Considering that a binary outcome was investigated (*L. monocytogenes* isolates were either susceptible or resistant), the effect size used to compare the prevalence of antimicrobial resistance to first- and second-line drugs was the risk difference. Initially, the risk of antimicrobial resistance in each group (first- and second-line drugs) was calculated by the equation:

(1) $$\begin{array}{c} \text{Risk}=\frac{\left(n_{\text{resistant isolates}}\right)}{n_{\text{analyzed isolates}}}. \end{array}$$

Then, the risk difference between the groups was calculated by the equation:

(2) $$\begin{array}{c} \text{Risk difference}=\left({\text{risk}}_{\text{second}-\text{line drugs}}\right)-\left({\text{risk}}_{\text{first}-\text{line drugs}}\right). \end{array}$$

Therefore, positive risk differences indicate higher prevalence of antimicrobial resistance in the second-line antimicrobials. Negative risk differences, higher prevalence of antimicrobial resistance in the first-line antimicrobials.

Individual standard errors for each comparison of groups were calculated by the equation:

(3) $$\begin{array}{c} SE=\frac{a_{i}Xb_{i}}{n_{i1}^{3}}+\frac{c_{i}Xd_{i}}{n_{i2}^{3}}, \end{array}$$

where *a*_*i*_ = number of *L. monocytogenes* isolates resistant to second-line drugs; *b*_*i*_ = number of *L. monocytogenes* isolates not resistant to second-line drugs; *c*_*i*_ = number of *L. monocytogenes* isolates resistant to first-line drugs; *d*_*i*_ = number of *L. monocytogenes* isolates not resistant to first-line drugs; *n*_*i*1_ = total number of *L. monocytogenes* isolates tested for second-line drugs; *n*_*i*2_ = total number of *L. monocytogenes* isolates tested for first-line drugs. With this information, confidence intervals (95%) were calculated and risk differences were pooled to estimate the overall risk difference, weighting studies contribution according to the inverse variance method. The significance (*p* < 0.05) of pooled risk differences was assessed by the *Z* test.

Total heterogeneity between studies was assessed by Tau^2^ and its significance (*p* < 0.10) was tested by the chi-square test. Heterogeneity in the true effect size (without considering sampling errors of individual studies) was estimated by the inconsistency index (*I*^2^). Total heterogeneity was incorporated into the meta-analysis by assigning random effect to the studies. In case of significant heterogeneity, subgroup analyses were performed using the classification items coded during the data extraction procedure.

Meta-analysis robustness was verified through sensitivity analysis. A funnel plot was generated by plotting individual risk differences against their standard errors. Publication bias (funnel plot asymmetry) was tested by Egger`s regression (*p* < 0.05) [51]. Potential outliers were detected by the visual interpretation of the funnel plot and their interference on the overall effect was checked by their exclusion from the dataset and re-analysis. When the presence of a potential outlier did not change the overall result, the study was kept in the meta-analysis. In the end, the summary of most relevant results and statistics was presented following the recommendations of the PRISMA statement [52].

## 3. Results and Discussion

From the total of 302 identified studies, 16 met all the eligibility criteria and were included in this meta-analysis (Figure 1). These studies comprised a total population of 1152 *L. monocytogenes* isolates, obtained from different animal food products (*n* = 572), food processing environment (*n* = 553), and live animals (*n* = 27) (Table 1). The included studies were developed in different parts of the world, such as South America (*n* = 5), Europe (*n* = 4), Asia (*n* = 3), Africa (*n* = 2), and North America (*n* = 2). The studies from Europe [9, 38, 44, 50] accounted for the largest number of *L. monocytogenes* isolates (*n* = 356), while those from Asia [37, 47] responded for the smallest amount (*n* = 128).

Disk diffusion was the most used method for testing antimicrobial susceptibility. It was adopted in eight different studies, comprising a total of 570 *L. monocytogenes* isolates tested. Microdilution was described in seven studies (*n* = 518), and the vitek-2, in two studies (*n* = 64).

A total of 35 antimicrobials were assessed in the whole dataset, 11 of them classified as first-line drugs (amikacin, amoxicillin, ampicillin, gentamicin, kanamycin, oxacillin, penicilin, streptomycin, sulfamethoxazole, sulfonamides, and trimethoprim). The antimicrobials tested in the higher number of isolates were tetracyclin (800 *L. monocytogenes* isolates in 10 different studies) and clindamycin (749 *L. monocytogenes* isolates in 8 different studies). Amoxicillin, benzylpenicillin, cefaclor, cefepime, cefoxitin, clarithromycin, enrofloxacin, fusidic acid, imipenem, nitrofurantoin, sulfamethoxazole, sulfonamides, and vancomycin were those tested in the lowest number of isolates (less than 50 *L. monocytogenes* isolates each) and, except for sulfonamides [41, 49], they were all evaluated in just one study.

Complete lack of antimicrobial resistance across the studies (all *L. monocytogenes* isolates tested susceptible) was only observed for linezolid, an oxazolidinone analyzed in two different studies [46, 50] in a total of 238 *L. monocytogenes* isolates obtained from chicken and beef production chains. The use of linezolid in the treatment of listeriosis, however, is currently negligible. On the other hand, overall antimicrobial resistance (all *L. monocytogenes* isolates tested resistant) was described for amoxicillin (14 *L. monocytogenes* isolates), benzylpenicillin (25 *L. monocytogenes* isolates), cefoxitin (6 *L. monocytogenes* isolates), fusidic acid (25 *L. monocytogenes* isolates), imipenem (25 *L. monocytogenes* isolates), sulfamethoxazole (15 *L. monocytogenes* isolates), and vancomycin (3 *L. monocytogenes* isolates). As already mentioned, all of these drugs were tested in just one study each.

**Figure 2:**
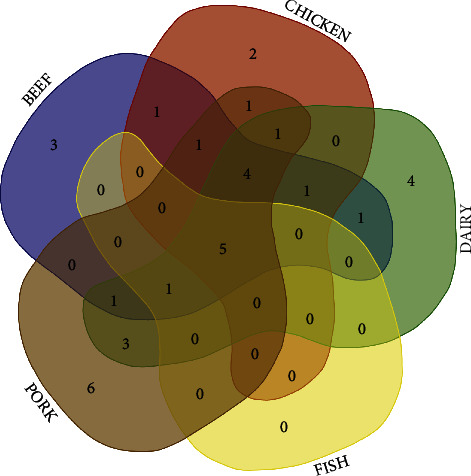
*Listeria monocytogenes* antimicrobial resistance across different animal production chains. PORK (*n* = 6): benzylpenicillin, cefotaxime, cephalosporin, doxycycline, fusidic acid, and imipenem. BEEF x CHICKEN x DAIRY x FISH x PORK (*n* = 5): ampicillin, clindamycin, erythromycin, tetracycline, trimethoprim. DAIRY (*n* = 4): amikacin, amoxicillin, cefaclor, and vancomycin. BEEF x CHICKEN x DAIRY x PORK (*n* = 4): chloramphenicol, ciprofloxacin, oxacillin, and sulfamethoxazole. BEEF (*n* = 3): cefepime, cefoxitin, and nalidixic acid. DAIRY x PORK (*n* = 3): fosfomycin, kanamycin, and streptomycin. CHICKEN (*n* = 2): enrofloxacin, nitrofurantoin. BEEF x DAIRY x FISH x PORK (*n* = 1): penicillin. BEEF x CHICKEN x DAIRY (*n* = 1): ceftriaxone. BEEF x CHICKEN x PORK (*n* = 1): amoxicillin. BEEF x DAIRY x PORK (*n* = 1): trimethoprim + sulfamethoxazole. CHICKEN x DAIRY x PORK (*n* = 1): gentamicin. BEEF x CHICKEN (*n* = 1): sulfonamides. BEEF x DAIRY (*n* = 1): quinupristin + dalfopristin. CHICKEN x PORK (*n* = 1): rifampicin.

With a different proposal of analysis, Figure 2 shows the distribution of antimicrobial resistance (one or more *L. monocytogenes* isolates tested resistant) between the animal production chains. Individually, swine and dairy were the production chains where resistance to a greater number of molecules was detected (*n* = 23 and 21, respectively). On the other hand, *L. monocytogenes* from fish showed resistance to the smallest number of molecules (*n* = 6). \ *L. monocytogenes* strains resistant to five drugs (ampicillin, clindamycin, erythromycin, tetracycline, and trimethoprim) disseminated through all animal chains were identified. Ampicillin and trimethoprim are considered first-line drugs for the treatment of listeriosis.

### 3.1. Overall Risk Difference of Antimicrobial Resistance

**Figure 3:**
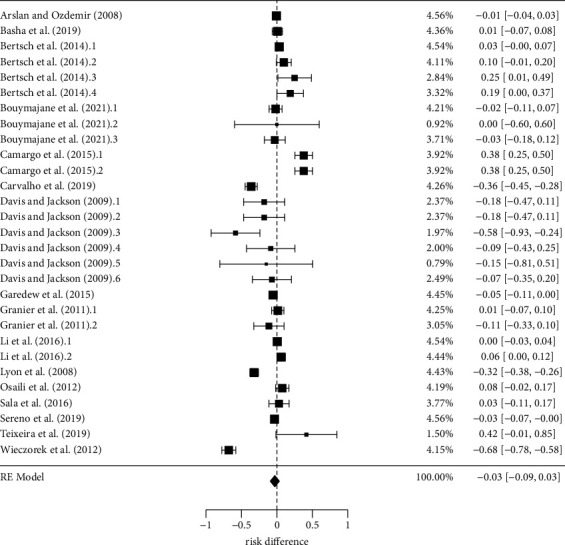
Overall risk difference of antimicrobial resistance to first- and second-line drugs in *Listeria monocytogenes*. Main estimates and statistics: *k* = 29; risk difference (*p*=0.37); Tau2 = 0.02; heterogeneity (*p* < 0.01); *I*^2^ = 94%.

An overall risk difference of 3% (−0.03 [−0.09, 0.03]) was estimated in the meta-analysis (Figure 3). However, this risk difference was not significant (*p*=0.37), meaning that there is no evidence that the prevalence of antimicrobial resistance in *L. monocytogenes* is different for first- and second-line antimicrobials. Significant heterogeneity (*p* < 0.01) was detected between studies and, based on the high value of the inconsistency index (*I*^2^ = 94%), the main hypothesis is that heterogeneity to originate mainly from their methodological differences, rather than sampling errors due to small number of *L. monocytogenes* isolates tested in each individual study. Therefore, subgroup analysis was performed aiming to explain at least a part of this heterogeneity and better discuss the intra-study characteristics that effectively contributed to the overall result.

**Figure 4:**
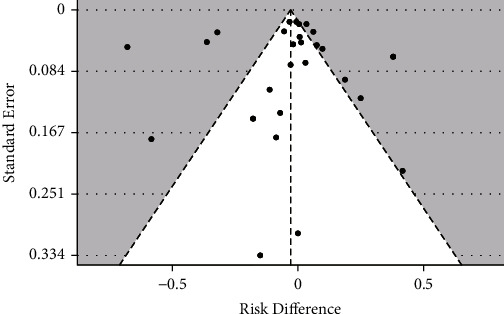
Funnel plot of individual risk differences against their standard errors. Egger's regression for funnel plot asymmetry (*p*=0.96).

The robustness of this overall risk difference estimate was high. First, no evidence of publication bias was observed within the dataset. This interpretation was based not only on the apparently equal distribution of individual risk differences on both sides of the funnel plot, but also on the high *p*-value obtained (*p*=0.96) in the test of funnel plot asymmetry (Figure 4). Second, although some of the individual risk differences fell outside the 95% confidence triangle, their exclusion from the meta-analysis did not significantly change the overall result. Finally, a significant number of individual risk differences was located in the upper region of the funnel, where only observations with low associated errors (greater contribution to the overall effect estimate) are found.

Before the presentation of the subgroup analyses, it is important to note that, although our dataset was not small, many other identified studies (*n* = 25) were not included here because they used *L. monocytogenes* isolated from sources other than animals, animal food products, or food processing environment (Figure 1, cause of rejection #1). Those studies were mostly (16/25) conducted with *L. monocytogenes* obtained from sick people (clinical isolates). This situation is in full agreement with the objectives of the present meta-analysis: to answer whether the prevalence of antimicrobial resistance in *L. monocytogenes* isolated from animal foods is higher for first- or second-line antimicrobials. Therefore, our results reflect the antimicrobial resistance profile of *L. monocytogenes* from animal foods and their production chains and must not be generalized to the entire *L. monocytogenes* population. That would be too bold a goal for a single meta-analysis.

### 3.2. Risk Difference of Antimicrobial Resistance by Study Location

**Figure 5:**
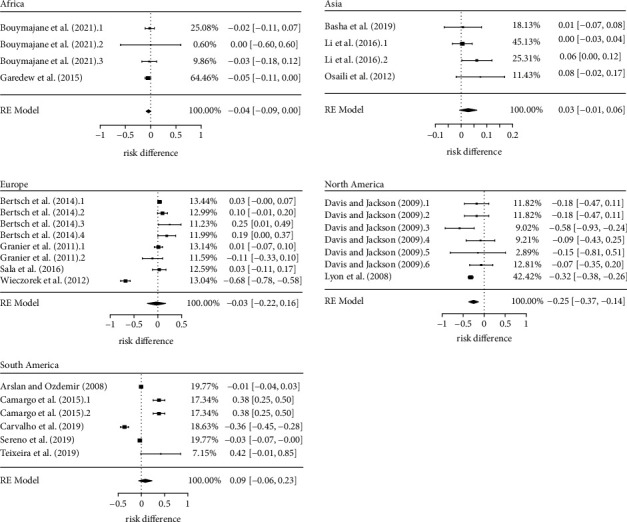
Risk difference of antimicrobial resistance to first- and second-line drugs in *Listeria monocytogenes* by study location.

The study location subgroup analysis aimed to answer whether the origin of *L. monocytogenes* isolates affects their antimicrobial resistance profile. The obtained results indicated that this is indeed the case (Figure 5). From all the continents assessed, only in the North America there was evidence that the risk of antimicrobial resistance in *L. monocytogenes* is higher (*p* < 0.01) for first- than second-line antimicrobials (Table 2). This higher risk was estimated in 25%. No significant risk differences were obtained in Africa (*p*=0.07), Asia (*p*=0.11), Europe (*p*=0.77), and South America (*p*=0.24).

Two studies [42] composed the dataset for the North America subgroup, both carried out in the United States. However, due intra-study differences regarding the animal production chain and *L. monocytogenes* source of isolation, David and Jackson [42] study was sliced into six comparison groups, thus totalizing 7 groups under this subgroup analysis (*k* = 7). The group with the greatest relevance (higher weight) to the composition of the pooled effect belonged to [46] accounting for 42.4% of the estimated pooled risk difference. This study was conducted with 157 *L. monocytogenes* isolates from chicken processing environment, the higher number of isolates assessed by an individual study among the 16 included in this meta-analysis. Certainly, this large sample size afforded a low variability to study's estimates, which led to its higher weight in the subgroup analysis [42] which assessed *L. monocytogenes* from beef, chicken, dairy, and pork, isolated in both live animals and their food products. A total of 10 antimicrobials were tested in the North America studies. Three of them (oxacillin, trimethoprim, and sulfamethoxazole) are considered as first-line drugs to the treatment of listeriosis.

Despite not achieving a significant *p*-value for the pooled risk difference, the proximity to a significant result obtained for Africa (*p* = 0.07) brings to this continent the need for further discussion. With an estimated 4% higher prevalence of antimicrobial resistance to first-line antimicrobials, Africa was also represented by two studies [39, 43], which composed 4 comparison groups to the subgroup analysis. Garedew et al. [43] accounted for 64.5% of the estimated polled risk difference.

This study was carried out in Ethiopia, using 140 *L. monocytogenes* isolates obtained from beef processing environment. With a much lower sampling size, [39] 14 *L. monocytogenes* isolates were assessed from beef, chicken, and dairy food products and their processing environment at Morocco. Together, these two studies tested *L. monocytogenes* resistance to 15 different antimicrobials, nine of them considered first-line antimicrobials to the treatment of listeriosis (amikacin, amoxicillin, ampicillin, gentamicin, kanamycin, penicillin, streptomycin, sulfamethoxazole, and trimethoprim). Lack of antimicrobial resistance was only described in [43], but not for all the isolates.

### 3.3. Risk Difference of Antimicrobial Resistance by Animal Production Chain

**Figure 6:**
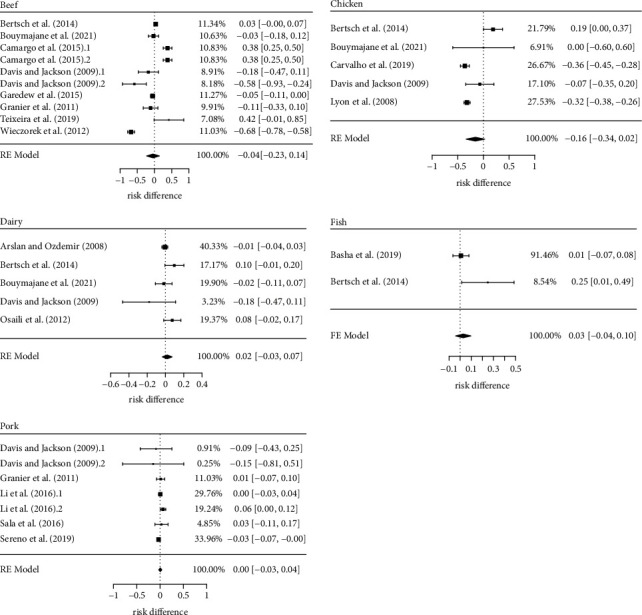
Risk difference of antimicrobial resistance to first- and second-line drugs in *Listeria monocytogenes* by animal production chain.

This subgroup analysis aimed to answer whether the profile of *L. monocytogenes* antimicrobial resistance is affected by production practices and other characteristics associated to the animal production chain they are isolated. Our results showed that there is no evidence in the literature to support this thesis (Figure 6).

Except for the chicken production chain, all the other *p*-values obtained from the pooled risk differences were much greater than 0.05 (Table 3). The beef subgroup was represented by eight studies (*k* = 10); chicken (*k* = 5), dairy (*k* = 5), and pork (*k* = 7) by five studies; and fish by two studies (*k* = 2). Due to the small number of observations, only the fixed-effect model fit the data from the fish subgroup analysis, which reduced the robustness of this specific estimative, as no random effect was attributed to the studies.

As mentioned before, the chicken production chain obtained the lower *p*-value (*p*=0.09) for the pooled risk difference, estimated at −0.16 (or 16%). It is worth to remember that negative risk differences indicate higher prevalence of antimicrobial resistance to first-line antimicrobials. In this subgroup, studies presented the analysis of *L. monocytogenes* isolated form chicken food products and their processing environment. Only the Asian continent was not represented in this subset of studies.

Resistance to seven first-line drugs and eleven second-line drugs was tested in the chicken subgroup. Only [42, 46] described complete lack of resistance to first-line drugs: oxacillin in the first study (*n* = 4) and trimethoprim + sulphamethoxazole in the second (*n* = 157). These same authors, however, described complete lack of resistance to a greater number of second-line drugs: ceftriaxone (*n* = 4), ciprofloxacin (*n* = 4), clindamycin (*n* = 161), quinupristin + dalfopristin (*n* = 4), and linezolid (*n* = 157).

### 3.4. Risk Difference of Antimicrobial Resistance by Source of Isolation

**Figure 7:**
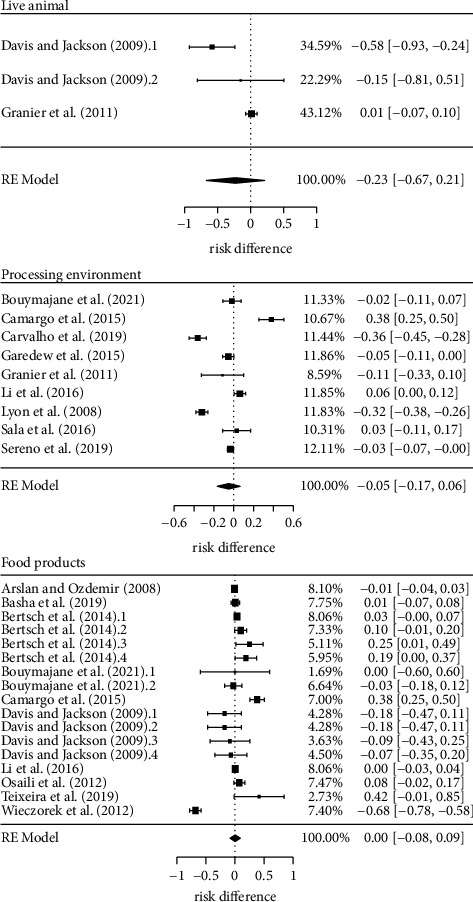
Risk difference of antimicrobial resistance to first- and second-line drugs in *Listeria monocytogenes* by source of isolation.

This last subgroup aimed to investigate whether the source of *L. monocytogenes* isolation is a significant determinant of its antimicrobial resistance profile. Our results demonstrated that there is no evidence in the literature that the prevalence of resistance to first- and second-line drugs is influenced by the source of *L. monocytogenes* isolation (Figure 7). Except for the live animal source (*n* = 2, *k* = 3), a good set of studies were obtained in all the subgroups. Even so, *p*-values for the pooled estimated risk difference were not lower than 0.31 (Table 4).

One important aspect to consider in all the subgroup analyses presented here is that, in most cases, significant heterogeneity persisted in the dataset even after their separation into groups of common characteristics such as study location, animal production chain, and *L. monocytogenes* source of isolation. More important, the I^2^ values were kept high in almost all the subgroups. In short, the *I*^2^ index expresses the amount of the total variance between studies (Tau^2^) that persisted in the dataset when the intra-study sampling error is not accounted. In other words, this index is an estimate of the range of methodological differences between studies in the subset under analysis. Therefore, our results showed that the published literature on *L. monocytogenes* phenotypic antimicrobial resistance is significantly diverse in terms of methodology. Unfortunately, not all of this diversity (populational, analytical and others) were comprehensively described in the included studies. Thus, much of this heterogeneity remained unexplained.

The strategy adopted in the present meta-analysis was not designed to allow for an objective interpretation or even an explanation for the causes of significant differences in the prevalence of resistance to first- and second-line antimicrobials. Nevertheless, because the use of antimicrobials in the animal production systems is regulated differently in each country, one could suggest that the finding of significant risk difference only in the United States might be associated with their politics on the use of antimicrobials. In fact, this is a serious question that deserves a proper analysis in another study, not only for the United States but for all the places where meaningful data are published. Probably, the addressment of this question exclusively in *L. monocytogenes* is currently limited by the few published studies in this area. But this is a much broader question than the one investigated here, and could be answered using different microorganisms, alone or in combination.

To the best of our knowledge, this study represents the first meta-analysis comparing the prevalence of resistance to first- and second-line antimicrobials in *L. monocytogenes* from animal foods. Although its robustness has been proven, a greater volume of publication, together with better characterization of the isolates, their origin, and the procedures used in the susceptibility assays, are still needed for a more precise estimate of the real prevalence of antimicrobial resistance in this population. In particular, more studies within the fish production chain are desirable.

## 4. Conclusions

Overall, there is no evidence in the literature that the prevalence of antimicrobial resistance in *Listeria monocytogenes* isolated from animal foods is higher for first- than second-line antimicrobials. However, this situation is different in the United States, as a 25% higher prevalence of resistance to first-line drugs has been estimated. Furthermore, there is no evidence that the animal production chain or the source of *Listeria monocytogenes* isolation affects its antimicrobial resistance profile. However, it is important to emphasize that, due to the small number of studies on fish concerning antimicrobial resistance of *L. monocytogenes*, the results in this animal production chain may be underestimated. Therefore, further research is encouraged using these animals, in order to achieve a more realistic scenario concerning *L. monocytogenes* resistance. Finally, the present study will be able to guide prevention and control measures for *L. monocytogenes* and contribute to the development of a One Health approach, associating concepts between veterinary medicine, human health, animal production systems, and the environment.

## Figures and Tables

**Table 1:** Summary of included studies.

Study	Country	*L. monocytogenes* isolates (*n*)	*L. monocytogenes* origin	Antimicrobial susceptibility testing	Resistance detected to (one or more *L. monocytogenes* isolate tested resistant)	No resistance detected to (all isolates tested susceptible)
[36]	Peru	47	Dairy (food product)	Disk diffusion	Amikacin, ampicillin, cefaclor, clarithromycin, chloramphenicol, penicillin, tetracycline	Ciprofloxacin, gentamycin, rifampicin, trimethoprim + sulfamethoxazole
[37]	India	11	Fish (food product)	Disk diffusion	Ampicillin, clindamycin, erythromycin, penicillin, tetracycline	—
[38]	Switzerland	220	Beef, chicken, dairy, fish (food product)	Microdilution	Clindamycin, tetracycline, trimethoprim,	—
[39]	Morocco	14	Beef, chicken, dairy (food product, processing environment)	Disk diffusion	Amikacin, amoxicillin, ampicillin, kanamycin, ciprofloxacin, chloramphenicol, erythromycin, streptomycin, gentamicin, sulfamethoxazole, tetracycline, trimethoprim + sulfamethoxazole, vancomycin	—
[40]	Brazil	156	Beef (food product, processing environment)	Disk diffusion	Clindamycin, oxacillin	—
[41]	Brazil	37	Chicken (processing environment)	Disk diffusion	Ampicillin, ciprofloxacin, chloramphenicol, enrofloxacin, erythromycin, gentamycin, nitrofurantoin, rifampicin, sulfonamides, trimethoprim	—
[42]	USA	24	Beef, chicken, dairy, pork (live animal, processing environment)	Microdilution	Ceftriaxone, ciprofloxacin, clindamycin, oxacillin, quinupristin + dalfopristin	—
[43]	Ethiopia	140	Beef (processing environment)	Disk diffusion	Chloramphenicol, nalidixic acid, penicillin, tetracycline	—
[44]	France	30	Beef, pork (live animal, processing environment)	Microdilution	Tetracycline, trimethoprim	Erythromycin
[45]	China	78	Pork (food product, processing environment)	Disk diffusion	Ampicillin, cephalosporin, cefotaxime, chloramphenicol, doxycycline, erythromycin, streptomycin, gentamycin, rifampicin, tetracycline, trimethoprim	—
[46]	USA	157	Chicken (processing environment)	Microdilution	Ceftriaxone, ciprofloxacin, oxacillin, tetracycline	Clindamycin, linezolid, trimethoprim + sulfamethoxazole
[47]	Jordan	39	Dairy (food product)	Vitek-2	Fosfomycin, oxacillin	—
[9]	Romania	25	Pork (processing environment)	Vitek-2	Benzylpenicillin, ciprofloxacin, clindamycin, fosfomycin, fusidic acid, imipenem, oxacillin, rifampicin, tetracycline, trimethoprim	—
[48]	Brazil	87	Pork (processing environment)	Disk diffusion	Ampicillin, kanamycin, clindamycin, erythromycin, penicillin, tetracycline, trimethoprim + sulfamethoxazole	—
[49]	Brazil	6	Beef (food product)	Microdilution	Cefoxitin, cefepime, sulfonamides	—
[50]	Poland	81	Beef (food product)	Microdilution	Ceftriaxone, ciprofloxacin, oxacillin, quinupristin + dalfopristin	Clindamycin, linezolid

*L. monocytogenes* = *Listeria monocytogenes*.

**Table 2:** Estimates and main statistics of the risk difference of antimicrobial resistance in *Listeria monocytogenes* by study location.

Study location	*k*	Risk difference		Heterogeneity		
		Estimate (CI, 95%)	*p*-value	Tau^2^	*p*-value	*I* ^2^ (%)
Africa	4	−0.04 (−0.09, 0.00)	0.07	<0.01	0.92	0
Asia	4	0.03 (−0.01, 0.06)	0.11	<0.01	0.28	22
Europe	8	−0.03 (−0.22, 0.16)	0.77	0.07	<0.01	96
North America	7	−0.25 (−0.37, −0.14)	<0.01	0.01	0.19	32
South America	6	0.09 (−0.06, 0.23)	0.24	0.03	<0.01	97

**Table 3:** Estimates and main statistics of the risk difference of antimicrobial resistance in *Listeria monocytogenes* by animal production chain.

Animal production chain	*k*	Risk difference		Heterogeneity		
		Estimate (CI, 95%)	*p*-value	Tau^2^	*p*-value	*I* ^2^ (%)
Beef	10	−0.04 (−0.23, 0.14)	0.65	0.08	<0.01	97
Chicken	5	−0.16 (−0.34, 0.02)	0.09	0.03	<0.01	88
Dairy	5	0.02 (−0.03, 0.07)	0.49	<0.01	0.11	47
Fish	2	0.03 (−0.04, 0.10)	0.43	0.00	0.05	73
Pork	7	0.01 (−0.03, 0.04)	0.83	<0.01	0.17	34

**Table 4:** Estimates and main statistics of the risk difference of antimicrobial resistance in *Listeria monocytogenes* by source of isolation.

*L. monocytogenes* source of isolation	*k*	Risk difference		Heterogeneity		
		Estimate (CI, 95%)	*p*-value	Tau^2^	*p*-value	*I* ^2^ (%)
Live animal	3	−0.23 (−0.67, 0.21)	0.31	0.12	0.93	93
Processing environment	9	−0.05 (−0.17, 0.06)	0.38	0.03	<0.01	96
Food product	17	0.01 (−0.08, 0.09)	0.93	0.02	<0.01	93

## Data Availability

The data used to support this study are provided as supplementary materials.

## Supplementary Materials

Supplementary Materials Table S1 was included showing the articles that were analyzed in this meta-analysis, as well as the antimicrobials used and their respective classifications as conventional or not and the number of resistant strains.
