# Supplementary material for: Antimicrobial Resistance of Listeria monocytogenes from Animal Foods to First- and Second-Line Drugs in the Treatment of Listeriosis from 2008 to 2021: A Systematic Review and Meta-Analysis
Source: Can J Infect Dis Med Microbiol. 2022 Oct 7;2022:1351983. doi: 10.1155/2022/1351983 (PMC9568363; doi:10.1155/2022/1351983)
Supplement: Supplementary Materials — Table S1 was included showing the articles that were analyzed in this meta-analysis, as well as the antimicrobials used and their respective classifications as conventional or not and the number of resistant strains. [file 1351983.f1.pdf]

Study	Year	Country	Continent	Animal Chain	Source of Isolation	Antibiotics	Class	METANALISIS	RESISTENT	TOTAL
Arslan et al. (2008)	2008	Peru	Asia	dairy	product	Cefaclor	Cefens	NON-CONVENTIONAL	1	47
Arslan et al. (2008)	2008	Peru	Asia	dairy	product	Clarithromycin	Macrolides	NON-CONVENTIONAL	3	47
Arslan et al. (2008)	2008	Peru	Asia	dairy	product	Tetracycline	Tetracyclines	NON-CONVENTIONAL	1	47
Arslan et al. (2008)	2008	Peru	Asia	dairy	product	Ciprofloxacin	Quinolones	NON-CONVENTIONAL	0	47
Arslan et al. (2008)	2008	Peru	Asia	dairy	product	Chloramphenicol	Phenicol	NON-CONVENTIONAL	4	47
Arslan et al. (2008)	2008	Peru	Asia	dairy	product	Rifampicin	Macrolides	NON-CONVENTIONAL	0	47
Arslan et al. (2008)	2008	Peru	Asia	dairy	product	Ampicillin	Penicillins	CONVENTIONAL	1	47
Arslan et al. (2008)	2008	Peru	Asia	dairy	product	Penicillin	Penicillins	CONVENTIONAL	6	47
Arslan et al. (2008)	2008	Peru	Asia	dairy	product	Amikacin	Aminoglycosides	CONVENTIONAL	2	47
Arslan et al. (2008)	2008	Peru	Asia	dairy	product	Gentamicin	Aminoglycosides	CONVENTIONAL	0	47
Arslan et al. (2008)	2008	Peru	Asia	dairy	product	Trimethoprim/Sulfamethoxazole	Folate inhibitors	CONVENTIONAL	0	47
Basha et al. (2019)	2019	India	Asia	fish	product	Erythromycin	Macrolides	NON-CONVENTIONAL	11	11
Basha et al. (2019)	2019	India	Asia	fish	product	Tetracycline	Tetracyclines	NON-CONVENTIONAL	11	11
Basha et al. (2019)	2019	India	Asia	fish	product	Clindamycin	lincosamides	NON-CONVENTIONAL	11	11
Basha et al. (2019)	2019	India	Asia	fish	product	Ampicillin	Penicillins	CONVENTIONAL	11	11
Basha et al. (2019)	2019	India	Asia	fish	product	Penicillin	Penicillins	CONVENTIONAL	11	11
Bertsch et al. (2014)	2014	Switzerland	Europe	dairy	product	Tetracycline	Tetracyclines	NON-CONVENTIONAL	11	46
Bertsch et al. (2014)	2014	Switzerland	Europe	fish	product	Tetracycline	Tetracyclines	NON-CONVENTIONAL	11	18
Bertsch et al. (2014)	2014	Switzerland	Europe	chicken	product	Tetracycline	Tetracyclines	NON-CONVENTIONAL	11	24
Bertsch et al. (2014)	2014	Switzerland	Europe	chicken	product	Clindamycin	lincosamides	NON-CONVENTIONAL	4	24
Bertsch et al. (2014)	2014	Switzerland	Europe	chicken	product	Trimethoprim	Folate inhibitors	CONVENTIONAL	3	24
Bertsch et al. (2014)	2014	Switzerland	Europe	beef	product	Tetracycline	Tetracyclines	NON-CONVENTIONAL	11	132
Bertsch et al. (2014)	2014	Switzerland	Europe	beef	product	Clindamycin	lincosamides	NON-CONVENTIONAL	4	132
Bertsch et al. (2014)	2014	Switzerland	Europe	dairy	product	Clindamycin	lincosamides	NON-CONVENTIONAL	4	46
Bertsch et al. (2014)	2014	Switzerland	Europe	fish	product	Clindamycin	lincosamides	NON-CONVENTIONAL	4	18
Bertsch et al. (2014)	2014	Switzerland	Europe	beef	product	Trimethoprim	Folate inhibitors	CONVENTIONAL	3	132
Bertsch et al. (2014)	2014	Switzerland	Europe	dairy	product	Trimethoprim	Folate inhibitors	CONVENTIONAL	3	46
Bertsch et al. (2014)	2014	Switzerland	Europe	fish	product	Trimethoprim	Folate inhibitors	CONVENTIONAL	3	18
Bouymajane et al. (2021)	2021	Marocco	Africa	whey milk	Environmental/processing	kanamycin	Aminoglycosides	CONVENTIONAL	3	3
Bouymajane et al. (2021)	2021	Marocco	Africa	whey milk	Environmental/processing	Vancomycin	Glycopeptides	NON-CONVENTIONAL	3	3
Bouymajane et al. (2021)	2021	Marocco	Africa	whey milk	Environmental/processing	Streptomycin	Aminoglycosides	CONVENTIONAL	3	3
Bouymajane et al. (2021)	2021	Marocco	Africa	whey milk	Environmental/processing	Amikacin	Aminoglycosides	CONVENTIONAL	3	3
Bouymajane et al. (2021)	2021	Marocco	Africa	whey milk	Environmental/processing	Gentamicin	Aminoglycosides	CONVENTIONAL	3	3
Bouymajane et al. (2021)	2021	Marocco	Africa	whey milk	Environmental/processing	Chloramphenicol	Phenicol	NON-CONVENTIONAL	3	3
Bouymajane et al. (2021)	2021	Marocco	Africa	whey milk	Environmental/processing	Ampicillin	Penicillins	CONVENTIONAL	3	3
Bouymajane et al. (2021)	2021	Marocco	Africa	whey milk	Environmental/processing	Sulfamethoxazole	Folate inhibitors	CONVENTIONAL	3	3
Bouymajane et al. (2021)	2021	Marocco	Africa	whey milk	Environmental/processing	Amoxicillin	β- lactams	CONVENTIONAL	3	3
Bouymajane et al. (2021)	2021	Marocco	Africa	whey milk	Environmental/processing	Erythromycin	Macrolides	NON-CONVENTIONAL	3	3
Bouymajane et al. (2021)	2021	Marocco	Africa	whey milk	Environmental/processing	Streptomycin	Aminoglycosides	CONVENTIONAL	3	3
Bouymajane et al. (2021)	2021	Marocco	Africa	raw milk	Environmental/processing	Ampicillin	Penicillins	CONVENTIONAL	3	3
Bouymajane et al. (2021)	2021	Marocco	Africa	raw milk	Environmental/processing	Amikacin	Aminoglycosides	CONVENTIONAL	3	3
Bouymajane et al. (2021)	2021	Marocco	Africa	raw milk	Environmental/processing	Erythromycin	Macrolides	NON-CONVENTIONAL	3	3
Bouymajane et al. (2021)	2021	Marocco	Africa	raw milk	Environmental/processing	Sulfamethoxazole	Folate inhibitors	CONVENTIONAL	3	3
Bouymajane et al. (2021)	2021	Marocco	Africa	raw milk	Environmental/processing	Trimethoprim/Sulfamethoxazole	Folate inhibitors	CONVENTIONAL	3	3
Bouymajane et al. (2021)	2021	Marocco	Africa	raw milk	Environmental/processing	Ciprofloxacin	Quinolones	NON-CONVENTIONAL	3	3
Bouymajane et al. (2021)	2021	Marocco	Africa	raw milk	Environmental/processing	Amoxicillin	β- lactams	CONVENTIONAL	3	3
Bouymajane et al. (2021)	2021	Marocco	Africa	chicken	product	Amoxicillin	β- lactams	CONVENTIONAL	1	1
Bouymajane et al. (2021)	2021	Marocco	Africa	chicken	product	Erythromycin	Macrolides	NON-CONVENTIONAL	1	1
Bouymajane et al. (2021)	2021	Marocco	Africa	chicken	product	Sulfamethoxazole	Folate inhibitors	CONVENTIONAL	1	1
Bouymajane et al. (2021)	2021	Marocco	Africa	chicken	product	Tetracycline	Tetracyclines	NON-CONVENTIONAL	1	1
Bouymajane et al. (2021)	2021	Marocco	Africa	beef	product	Amoxicillin	β- lactams	CONVENTIONAL	4	4
Bouymajane et al. (2021)	2021	Marocco	Africa	beef	product	Erythromycin	Macrolides	NON-CONVENTIONAL	4	4
Bouymajane et al. (2021)	2021	Marocco	Africa	beef	product	Sulfamethoxazole	Folate inhibitors	CONVENTIONAL	4	4
Bouymajane et al. (2021)	2021	Marocco	Africa	beef	product	Tetracycline	Tetracyclines	NON-CONVENTIONAL	4	4
Bouymajane et al. (2021)	2021	Marocco	Africa	beef	product	Ampicillin	Penicillins	CONVENTIONAL	4	4
Bouymajane et al. (2021)	2021	Marocco	Africa	beef	product	Erythromycin	Macrolides	NON-CONVENTIONAL	1	1
Bouymajane et al. (2021)	2021	Marocco	Africa	beef	product	Sulfamethoxazole	Folate inhibitors	CONVENTIONAL	1	1
Bouymajane et al. (2021)	2021	Marocco	Africa	beef	product	Trimethoprim/Sulfamethoxazole	Folate inhibitors	CONVENTIONAL	1	1

Bouymajane et al. (2021)	2021	Marocco	Africa	chicken	product	Amoxicillin	β- lactams	CONVENTIONAL	3	3
Bouymajane et al. (2021)	2021	Marocco	Africa	chicken	product	Sulfamethoxazole	Folate inhibitors	CONVENTIONAL	3	3
Bouymajane et al. (2021)	2021	Marocco	Africa	chicken	product	Trimethoprim/Sulfamethoxazole	Folate inhibitors	CONVENTIONAL	3	3
Camargo et al. (2015)	2015	Brazil	South America	beef	Environmental	Clindamycin	lincosamides	NON-CONVENTIONAL	67	72
Camargo et al. (2015)	2015	Brazil	South America	beef	Environmental	Oxacillin	Penicillins	CONVENTIONAL	43	78
Camargo et al. (2015)	2015	Brazil	South America	beef	product	Clindamycin	lincosamides	NON-CONVENTIONAL	67	72
Camargo et al. (2015)	2015	Brazil	South America	beef	product	Oxacillin	Penicillins	CONVENTIONAL	43	78
Carvalho et al. (2019)	2019	Brazil	South America	chicken	Environmental	Ciprofloxacin	Fluoroquinolones	NON-CONVENTIONAL	3	37
Carvalho et al. (2019)	2019	Brazil	South America	chicken	Environmental	Enrofloxacin	Fluoroquinolones	NON-CONVENTIONAL	1	37
Carvalho et al. (2019)	2019	Brazil	South America	chicken	Environmental	Nitrofurantoin	Nitrofuran	NON-CONVENTIONAL	7	37
Carvalho et al. (2019)	2019	Brazil	South America	chicken	Environmental	Rifampicin	Ansamycin	NON-CONVENTIONAL	1	37
Carvalho et al. (2019)	2019	Brazil	South America	chicken	Environmental	Chloramphenicol	Phenicol	NON-CONVENTIONAL	1	37
Carvalho et al. (2019)	2019	Brazil	South America	chicken	Environmental	Erythromycin	Macrolides	NON-CONVENTIONAL	1	37
Carvalho et al. (2019)	2019	Brazil	South America	chicken	Environmental	Sulfonamides	Folate inhibitors	CONVENTIONAL	34	37
Carvalho et al. (2019)	2019	Brazil	South America	chicken	Environmental	Trimethoprim	Folate inhibitors	CONVENTIONAL	1	37
Carvalho et al. (2019)	2019	Brazil	South America	chicken	Environmental	Ampicillin	Penicillins	CONVENTIONAL	25	37
Carvalho et al. (2019)	2019	Brazil	South America	chicken	Environmental	Gentamicin	Aminoglycosides	CONVENTIONAL	3	37
Davis et al. (2009)	2009	United States	North America	beef	animal	Clindamycin	lincosamides	NON-CONVENTIONAL	6	6
Davis et al. (2009)	2009	United States	North America	beef	animal	Ciprofloxacin	Quinolones	NON-CONVENTIONAL	0	6
Davis et al. (2009)	2009	United States	North America	beef	animal	Ceftriaxone	Cefens	NON-CONVENTIONAL	0	6
Davis et al. (2009)	2009	United States	North America	beef	animal	Quinupristine / Dalfopristin	Streptogramins	NON-CONVENTIONAL	0	6
Davis et al. (2009)	2009	United States	North America	pork	animal	Clindamycin	lincosamides	NON-CONVENTIONAL	0	1
Davis et al. (2009)	2009	United States	North America	pork	animal	Ciprofloxacin	Quinolones	NON-CONVENTIONAL	0	1
Davis et al. (2009)	2009	United States	North America	pork	animal	Ceftriaxone	Cefens	NON-CONVENTIONAL	0	1
Davis et al. (2009)	2009	United States	North America	pork	animal	Quinupristine / Dalfopristin	Streptogramins	NON-CONVENTIONAL	0	1
Davis et al. (2009)	2009	United States	North America	beef	animal	Oxacillin	Penicillins	CONVENTIONAL	5	6
Davis et al. (2009)	2009	United States	North America	pork	animal	Oxacillin	Penicillins	CONVENTIONAL	0	1
Davis et al. (2009)	2009	United States	North America	dairy	product	Clindamycin	lincosamides	NON-CONVENTIONAL	0	5
Davis et al. (2009)	2009	United States	North America	dairy	product	Ciprofloxacin	Quinolones	NON-CONVENTIONAL	5	5
Davis et al. (2009)	2009	United States	North America	dairy	product	Ceftriaxone	Cefens	NON-CONVENTIONAL	5	5
Davis et al. (2009)	2009	United States	North America	dairy	product	Quinupristine / Dalfopristin	Streptogramins	NON-CONVENTIONAL	5	5
Davis et al. (2009)	2009	United States	North America	beef	product	Clindamycin	lincosamides	NON-CONVENTIONAL	0	5
Davis et al. (2009)	2009	United States	North America	beef	product	Ciprofloxacin	Quinolones	NON-CONVENTIONAL	5	5
Davis et al. (2009)	2009	United States	North America	beef	product	Ceftriaxone	Cefens	NON-CONVENTIONAL	5	5
Davis et al. (2009)	2009	United States	North America	beef	product	Quinupristine / Dalfopristin	Streptogramins	NON-CONVENTIONAL	5	5
Davis et al. (2009)	2009	United States	North America	pork	product	Clindamycin	lincosamides	NON-CONVENTIONAL	0	3
Davis et al. (2009)	2009	United States	North America	pork	product	Ciprofloxacin	Quinolones	NON-CONVENTIONAL	0	3
Davis et al. (2009)	2009	United States	North America	pork	product	Ceftriaxone	Cefens	NON-CONVENTIONAL	0	3
Davis et al. (2009)	2009	United States	North America	pork	product	Quinupristine / Dalfopristin	Streptogramins	NON-CONVENTIONAL	0	3
Davis et al. (2009)	2009	United States	North America	chicken	product	Clindamycin	lincosamides	NON-CONVENTIONAL	0	4
Davis et al. (2009)	2009	United States	North America	chicken	product	Ciprofloxacin	Quinolones	NON-CONVENTIONAL	0	4
Davis et al. (2009)	2009	United States	North America	chicken	product	Ceftriaxone	Cefens	NON-CONVENTIONAL	0	4
Davis et al. (2009)	2009	United States	North America	chicken	product	Quinupristine / Dalfopristin	Streptogramins	NON-CONVENTIONAL	0	4
Davis et al. (2009)	2009	United States	North America	dairy	product	Oxacillin	Penicillins	CONVENTIONAL	5	5
Davis et al. (2009)	2009	United States	North America	beef	product	Oxacillin	Penicillins	CONVENTIONAL	5	5
Davis et al. (2009)	2009	United States	North America	pork	product	Oxacillin	Penicillins	CONVENTIONAL	0	3
Davis et al. (2009)	2009	United States	North America	chicken	product	Oxacillin	Penicillins	CONVENTIONAL	0	4
Garedew et al. (2015)	2015	Ethiopia	Africa	beef	Environmental	Chloramphenicol	Phenicol	NON-CONVENTIONAL	4	140
Garedew et al. (2015)	2015	Ethiopia	Africa	beef	Environmental	Nalidixic acid	Quinolones	NON-CONVENTIONAL	12	140
Garedew et al. (2015)	2015	Ethiopia	Africa	beef	Environmental	Tetracycline	Tetracyclines	NON-CONVENTIONAL	9	140
Garedew et al. (2015)	2015	Ethiopia	Africa	beef	Environmental	Penicillin	Penicillins	CONVENTIONAL	16	140
Granier et al. (2011)	2011	France	Europe	beef	Environmental	Erythromycin	Macrolides	NON-CONVENTIONAL	0	10
Granier et al. (2011)	2011	France	Europe	beef	Environmental	Tetracycline	Tetracyclines	NON-CONVENTIONAL	0	10
Granier et al. (2011)	2011	France	Europe	beef	Environmental	Trimethoprim	Folate inhibitors	CONVENTIONAL	1	10
Granier et al. (2011)	2011	France	Europe	pork	animal	Erythromycin	Macrolides	NON-CONVENTIONAL	0	20
Granier et al. (2011)	2011	France	Europe	pork	animal	Tetracycline	Tetracyclines	NON-CONVENTIONAL	1	20
Granier et al. (2011)	2011	France	Europe	pork	animal	Trimethoprim	Folate inhibitors	CONVENTIONAL	0	20
Lili et al. (2016)	2016	China	Asia	pork	Environmental	Cephalosporin	Cefens	NON-CONVENTIONAL	0	13
Lili et al. (2016)	2016	China	Asia	pork	Environmental	Cefotaxime	Cefens	NON-CONVENTIONAL	0	13

Lili et al. (2016)	2016	China	Asia	pork	Environmental	Tetracycline	Tetracyclines	NON-CONVENTIONAL	6	13
Lili et al. (2016)	2016	China	Asia	pork	Environmental	Doxycycline	Tetracyclines	NON-CONVENTIONAL	0	13
Lili et al. (2016)	2016	China	Asia	pork	Environmental	Erythromycin	Macrolides	NON-CONVENTIONAL	0	13
Lili et al. (2016)	2016	China	Asia	pork	Environmental	Rifampicin	Macrolides	NON-CONVENTIONAL	0	13
Lili et al. (2016)	2016	China	Asia	pork	Environmental	Chloramphenicol	Phenicol	NON-CONVENTIONAL	0	13
Lili et al. (2016)	2016	China	Asia	pork	Environmental	Ampicillin	Penicillins	CONVENTIONAL	0	13
Lili et al. (2016)	2016	China	Asia	pork	Environmental	Streptomycin	Aminoglycosides	CONVENTIONAL	0	13
Lili et al. (2016)	2016	China	Asia	pork	Environmental	Gentamicin	Aminoglycosides	CONVENTIONAL	0	13
Lili et al. (2016)	2016	China	Asia	pork	Environmental	Trimethoprim	Folate inhibitors	CONVENTIONAL	0	13
Lili et al. (2016)	2016	China	Asia	pork	product	Cephalosporin	Cefens	NON-CONVENTIONAL	3	65
Lili et al. (2016)	2016	China	Asia	pork	product	Cefotaxime	Cefens	NON-CONVENTIONAL	6	65
Lili et al. (2016)	2016	China	Asia	pork	product	Tetracycline	Tetracyclines	NON-CONVENTIONAL	10	65
Lili et al. (2016)	2016	China	Asia	pork	product	Doxycycline	Tetracyclines	NON-CONVENTIONAL	4	65
Lili et al. (2016)	2016	China	Asia	pork	product	Erythromycin	Macrolides	NON-CONVENTIONAL	4	65
Lili et al. (2016)	2016	China	Asia	pork	product	Rifampicin	Macrolides	NON-CONVENTIONAL	3	65
Lili et al. (2016)	2016	China	Asia	pork	product	Chloramphenicol	Phenicol	NON-CONVENTIONAL	2	65
Lili et al. (2016)	2016	China	Asia	pork	product	Ampicillin	Penicillins	CONVENTIONAL	1	65
Lili et al. (2016)	2016	China	Asia	pork	product	Streptomycin	Aminoglycosides	CONVENTIONAL	7	65
Lili et al. (2016)	2016	China	Asia	pork	product	Gentamicin	Aminoglycosides	CONVENTIONAL	5	65
Lili et al. (2016)	2016	China	Asia	pork	product	Trimethoprim	Folate inhibitors	CONVENTIONAL	4	65
Lyon et al. (2008)	2008	United States	North America	chicken	Environmental	Ceftriaxone	Cefens	NON-CONVENTIONAL	93	157
Lyon et al. (2008)	2008	United States	North America	chicken	Environmental	Ciprofloxacin	Quinolones	NON-CONVENTIONAL	5	157
Lyon et al. (2008)	2008	United States	North America	chicken	Environmental	Clindamycin	lincosamides	NON-CONVENTIONAL	0	157
Lyon et al. (2008)	2008	United States	North America	chicken	Environmental	Tetracycline	Tetracyclines	NON-CONVENTIONAL	5	157
Lyon et al. (2008)	2008	United States	North America	chicken	Environmental	Linezolid	Oxazolidinone	NON-CONVENTIONAL	0	157
Lyon et al. (2008)	2008	United States	North America	chicken	Environmental	Oxacillin	Penicillins	CONVENTIONAL	142	157
Lyon et al. (2008)	2008	United States	North America	chicken	Environmental	Trimethoprim/Sulfamethoxazole	Folate inhibitors	CONVENTIONAL	0	157
Osaili et al. (2012)	2012	Jordan	Asia	dairy	product	Phosphomycin	Phosphomycins	NON-CONVENTIONAL	39	39
Osaili et al. (2012)	2012	Jordan	Asia	dairy	product	Oxacillin	Penicillins	CONVENTIONAL	36	39
Sala et al. (2016)	2016	Romania	Europe	pork	Environmental	Benzylpenicillin	Oxazolidinone	NON-CONVENTIONAL	25	25
Sala et al. (2016)	2016	Romania	Europe	pork	Environmental	Imipenem	Carbapenems	NON-CONVENTIONAL	25	25
Sala et al. (2016)	2016	Romania	Europe	pork	Environmental	Fusidic Acid	Triterpenoids	NON-CONVENTIONAL	25	25
Sala et al. (2016)	2016	Romania	Europe	pork	Environmental	Phosphomycin	Phosphomycins	NON-CONVENTIONAL	23	25
Sala et al. (2016)	2016	Romania	Europe	pork	Environmental	Clindamycin	lincosamides	NON-CONVENTIONAL	22	25
Sala et al. (2016)	2016	Romania	Europe	pork	Environmental	Rifampicin	Macrolides	NON-CONVENTIONAL	14	25
Sala et al. (2016)	2016	Romania	Europe	pork	Environmental	Tetracycline	Tetracyclines	NON-CONVENTIONAL	11	25
Sala et al. (2016)	2016	Romania	Europe	pork	Environmental	Ciprofloxacin	Quinolones	NON-CONVENTIONAL	1	25
Sala et al. (2016)	2016	Romania	Europe	pork	Environmental	Oxacillin	Penicillins	CONVENTIONAL	23	25
Sala et al. (2016)	2016	Romania	Europe	pork	Environmental	Trimethoprim	Folate inhibitors	CONVENTIONAL	12	25
Sereno et al. (2019)	2019	Brazil	South America	pork	Environmental	Clindamycin	lincosamides	NON-CONVENTIONAL	5	87
Sereno et al. (2019)	2019	Brazil	South America	pork	Environmental	Tetracycline	Tetracyclines	NON-CONVENTIONAL	1	87
Sereno et al. (2019)	2019	Brazil	South America	pork	Environmental	Erythromycin	Macrolides	NON-CONVENTIONAL	1	87
Sereno et al. (2019)	2019	Brazil	South America	pork	Environmental	kanamycin	Aminoglycosides	CONVENTIONAL	1	87
Sereno et al. (2019)	2019	Brazil	South America	pork	Environmental	Ampicillin	Penicillins	CONVENTIONAL	16	87
Sereno et al. (2019)	2019	Brazil	South America	pork	Environmental	Penicillin	Penicillins	CONVENTIONAL	3	87
Sereno et al. (2019)	2019	Brazil	South America	pork	Environmental	Trimethoprim/Sulfamethoxazole	Folate inhibitors	CONVENTIONAL	1	87
Teixeira et al. (2019)	2019	Brazil	South America	beef	product	Cefoxitin	Cephalosporin	NON-CONVENTIONAL	6	6
Teixeira et al. (2019)	2019	Brazil	South America	beef	product	Cefepime	Cephalosporin	NON-CONVENTIONAL	5	6
Teixeira et al. (2019)	2019	Brazil	South America	beef	product	Sulfonamides	Folate inhibitors	CONVENTIONAL	3	6
Wieczorek et al. (2012)	2012	Poland	Europe	beef	product	ceftriaxione	Cefens	NON-CONVENTIONAL	14	81
Wieczorek et al. (2012)	2012	Poland	Europe	beef	product	Clindamycin	lincosamides	NON-CONVENTIONAL	0	81
Wieczorek et al. (2012)	2012	Poland	Europe	beef	product	Ciprofloxacin	Quinolones	NON-CONVENTIONAL	3	81
Wieczorek et al. (2012)	2012	Poland	Europe	beef	product	Quinupristine / Dalfopristin	Streptogramins	NON-CONVENTIONAL	3	81
Wieczorek et al. (2012)	2012	Poland	Europe	beef	product	Linezolid	Penicillins	NON-CONVENTIONAL	0	81
Wieczorek et al. (2012)	2012	Poland	Europe	beef	product	Oxacillin	Penicillins	CONVENTIONAL	59	81
